# Artificial Intelligence in Venous Thromboembolism Prevention: A Narrative Review of Machine Learning, Deep Learning, and Natural Language Processing

**DOI:** 10.3390/jcdd13030119

**Published:** 2026-03-06

**Authors:** Daniela Nicoleta Crisan, Talida Georgiana Cut, Lucian-Flavius Herlo, Nina Ivanovic, Alexandra Herlo, Luana Alexandrescu, Andreea Sălcudean, Raluca Dumache

**Affiliations:** 1 Doctoral School, Victor Babes University of Medicine and Pharmacy Timisoara, 300041 Timisoara, Romania; daniela.crisan@umft.ro (D.N.C.); flavius.herlo@umft.ro (L.-F.H.); nina.ivanovic@umft.ro (N.I.); 2 Department XIII, Discipline of Infectious Diseases, Victor Babes University of Medicine and Pharmacy Timisoara, 2 Eftimie Murgu Square, 300041 Timisoara, Romania; talida.cut@umft.ro; 3 Department of Dermatology, Victor Babes University of Medicine and Pharmacy Timisoara, E. Murgu Square, Nr. 2, 300041 Timisoara, Romania; 4 Department of Ethics and Social Sciences, George Emil Palade University of Medicine, Pharmacy, Science and Technology of Târgu Mureş, 540139 Târgu Mureş, Romania; andreea.salcudean@umfst.ro; 5 Gastroenterology Department, “Sf. Apostol Andrei” Emergency County Hospital, 145 Tomis Blvd., 900591 Constanta, Romania; 6 Department of Forensic Medicine, Bioethics, Medical Ethics and Medical Law, Victor Babes University of Medicine and Pharmacy Timisoara, 300041 Timisoara, Romania; raluca.dumache@umft.ro; 7 Center for Ethics in Human Genetic Identifications, Victor Babes University of Medicine and Pharmacy Timisoara, E. Murgu Square, Nr. 2, 300041 Timisoara, Romania

**Keywords:** venous thromboembolism, deep vein thrombosis, pulmonary embolism, VTE prevention, artificial intelligence, machine learning, deep learning, neural networks

## Abstract

Venous thromboembolism (VTE), which includes deep vein thrombosis and pulmonary embolism, is a significant and preventable cause of morbidity and mortality worldwide. Despite the existence of clinical prediction models, biomarker-based risk assessments, and imaging techniques, gaps remain in accurately identifying and managing high-risk patients. In recent years, artificial intelligence has emerged as a transformative tool in healthcare, offering promising applications for enhancing VTE prevention strategies. This narrative review synthesizes current evidence on the use of artificial intelligence (AI) technologies including machine learning (ML), deep learning (DL), and natural language processing (NLP). We explore how supervised ML algorithms, such as random forests, support vector machines, and gradient boosting, improve predictive performance compared to traditional models by capturing complex, nonlinear relationships within electronic health record data. We also examine the role of DL models, particularly convolutional neural networks, in interpreting imaging data, achieving diagnostic accuracies comparable to expert radiologists. Additionally, the review highlights NLP applications in extracting risk-relevant information from unstructured clinical notes and the emerging integration of wearable device data and time-series analysis for dynamic risk assessment. We argue that the successful integration of AI into routine VTE prevention workflows requires rigorous prospective validation, cross-institutional collaboration, and thoughtful implementation into clinical decision support systems.

## 1. Introduction

**Figure 1:**
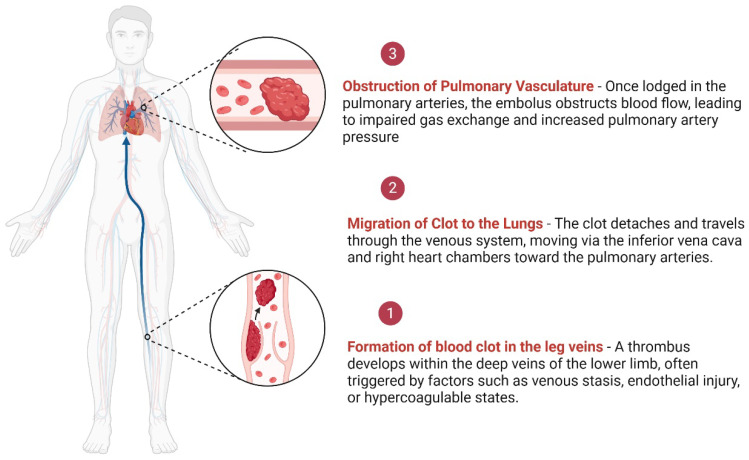
Pathway of pulmonary embolism formation. Created in BioRender. Crisan, D. (2026) [4].

Venous thromboembolism, encompassing deep vein thrombosis and pulmonary embolism, occurs with increased frequency in the context of obesity, surgical interventions, and malignancy, contributing substantially to preventable morbidity and mortality [1]. Obesity is associated with a two- to threefold increase in VTE risk, with incidence rising progressively with higher body mass index. Surgical interventions, particularly major orthopedic, abdominal, and oncologic procedures, account for approximately 40–60% of hospital-associated VTE events, despite routine thromboprophylaxis [2]. Malignancy represents another major risk factor, as patients with cancer experience a four- to sevenfold higher incidence of VTE compared with the general population, and cancer-associated thrombosis accounts for nearly 20% of all VTE cases worldwide [3]. Current clinical guidelines advocate for individualized VTE risk assessment, and existing preventive strategies include provider education, standardized medical admission order sets incorporating thromboprophylaxis, and electronic alert systems for patients identified as high risk [2]. Nevertheless, despite these recommendations, VTE prophylaxis remains inconsistently applied, and significant gaps persist in the diagnostic and therapeutic management of affected patients. Figure 1 shows the pathway to embolism formation.

Artificial intelligence and machine learning remain underutilized approaches in the prevention and management of venous thromboembolism. Owing to their versatility and scalability, AI/ML methods can circumvent several assumptions inherent to traditional statistical models, such as predefined error distributions and proportional hazards, rendering them particularly suitable for risk stratification, diagnostic support, and survival prediction [3]. ML algorithms are capable of generating forecasts based on heterogeneous data sources, including laboratory results, imaging findings, and unstructured physician documentation. Principal ML applications in the VTE domain include machine vision techniques for imaging-based VTE detection, natural language processing for identifying VTE diagnoses within medical records, and predictive modeling for individualized thrombotic risk estimation [5].

In the context of venous thromboembolism, artificial intelligence applications can be broadly categorized into three clinically distinct domains: risk prediction, diagnostic detection, and prevention-oriented decision support. Although these domains are interrelated, they differ substantially in their clinical objectives, evidentiary requirements, and implementation challenges, and should therefore be considered separately.

AI-based risk prediction models are designed to identify patients at increased likelihood of developing VTE before the occurrence of a clinical event. These tools typically operate upstream in the clinical workflow and rely on structured and unstructured data derived from electronic health records, laboratory values, and longitudinal patient characteristics. Their primary clinical value lies in stratifying patients for intensified surveillance or targeted thromboprophylaxis, rather than establishing a definitive diagnosis.

In contrast, diagnostic AI applications focus on the detection or confirmation of established VTE events, most commonly through imaging-based deep learning models applied to computed tomography pulmonary angiography or venous ultrasonography, as well as natural language processing systems applied to radiology reports. These tools function as diagnostic support systems and are subject to stringent accuracy, safety, and regulatory standards, given their direct impact on treatment decisions.

Finally, AI-driven prevention workflows represent downstream, implementation-focused applications that integrate predictive outputs into clinical decision support systems to guide thromboprophylaxis strategies in real time. These systems depend not only on predictive accuracy but also on seamless integration into electronic health records, clinician acceptance, and demonstrable impact on patient-centered outcomes. Importantly, while predictive and diagnostic models may inform prevention strategies, evidence supporting improved clinical outcomes through AI-guided VTE prevention remains limited and largely confined to early implementation or pilot studies.

Recent meta-analyses suggest that AI/ML technologies offer meaningful benefits in VTE-related applications; however, their translation into routine clinical practice remains limited, underscoring the need for further high-quality research and validation [6]. In this context, we have previously examined physicians’ acceptance and perspectives regarding the use of AI/ML in VTE prevention and management [4]. Notably, the effective deployment of advanced computational technologies requires interdisciplinary collaboration and domain-specific expertise, including the involvement of AI/ML informaticians.

**Figure 2:**
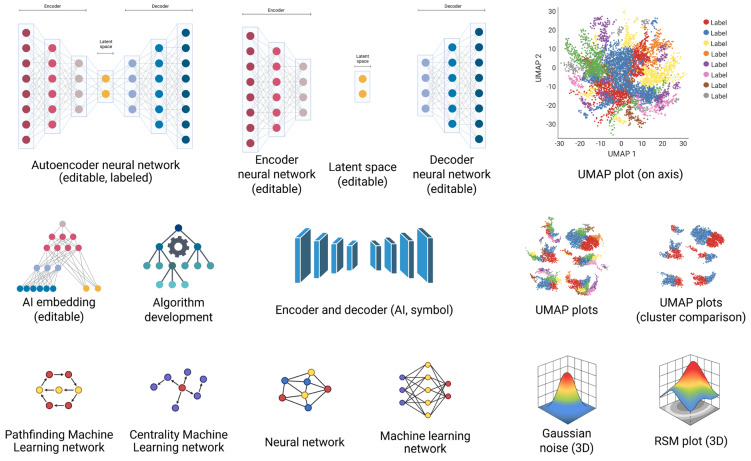
AI data and process illustration. Created in BioRender. Crisan, D. (2026) [3].

Clinical risk prediction models for VTE diagnosis and prognosis have been developed across a range of clinical settings, with model performance varying according to patient demographics, baseline thrombotic risk, and selected predictors [7]. A key limitation of conventional regression-based approaches, such as logistic and Cox regression, is their reliance on well-structured and carefully curated predictor variables [8]. Consequently, AI- and ML-based modeling techniques have gained popularity for prediction model development. Although these methods offer theoretical advantages, including computational flexibility and consistency, they are often affected by bias and limited interpretability, which hampers clinical acceptance and widespread implementation [9]. Moreover, AI-based models have not consistently demonstrated superior predictive performance when compared with traditional statistical approaches. In recent years, AI or machine learning-based VTE diagnosis and prognosis models have gained support. Supervised ML models (random forests, support vector machines, and gradient boosting), unsupervised approaches (for phenotyping and clustering), and, more recently, deep neural networks have been investigated for VTE tasks [10,11]. In addition, natural language processing (NLP) may extract clinical information from unstructured EHR notes, radiology reports, and discharge summaries, creating more complete patient profiles (Figure 2) [12].

The integration of AI into VTE prevention workflows raises several methodological, operational, and ethical challenges. Key issues currently under investigation include model interpretability, often referred to as the “black box” problem, data quality and representativeness, algorithmic bias, regulatory compliance, and effective clinical integration [13].

While several recent reviews have addressed the application of AI and machine learning in VTE and thrombosis, most have focused predominantly on algorithmic development, diagnostic accuracy, or isolated clinical use cases. In contrast, the present narrative review aims to provide an integrative, prevention-oriented perspective that explicitly bridges methodological innovation with clinical applicability. Rather than evaluating AI performance in isolation, we contextualize predictive, diagnostic, and decision-support tools within real-world VTE prevention workflows, emphasizing their translational readiness, implementation challenges, and clinical relevance.

Unlike prior reviews that predominantly focus on algorithmic development or isolated diagnostic applications, the present narrative review adopts an explicitly prevention-oriented and workflow-centered perspective. We introduce a structured conceptual framework that clearly distinguishes among AI applications for risk prediction, diagnostic detection, and prevention-oriented clinical decision support, recognizing that these domains differ substantially in their clinical objectives, evidentiary requirements, and implementation barriers. Furthermore, rather than evaluating model performance metrics in isolation, we contextualize AI tools within real-world VTE prevention pathways, emphasizing integration into clinical workflows, decision support environments, and outcome-driven validation.

## 2. Materials and Methods

This narrative review was conducted to explore, synthesize, and critically assess the current landscape of artificial intelligence applications in the prevention of venous thromboembolism. Given the interdisciplinary nature of the topic, we employed a broad and flexible search approach, focusing on both foundational and emerging literature across the fields of thrombosis medicine, predictive analytics, machine learning, and health informatics.

### 2.1. Literature Sources

Relevant publications were identified through searches of electronic databases, including PubMed, Scopus, Web of Science, IEEE Xplore, and Google Scholar, covering the period from January 2010 to December 2025. We included peer-reviewed original research articles, landmark trials, high-impact reviews, expert commentaries, and selected conference proceedings. To ensure comprehensive coverage, additional sources were identified by manually screening the reference lists of key papers and recent reviews.

### 2.2. Search Focus

No formal language or publication-type restrictions were imposed, but priority was given to English-language studies and those with substantial methodological detail.

### 2.3. Selection Approach

Unlike systematic reviews, narrative reviews do not follow rigid inclusion/exclusion protocols. Instead, we prioritized studies and reports based on relevance, methodological innovation, and potential clinical impact. Studies were selected if they contributed meaningful insights into:

- The design and development of AI-based diagnostic or predictive models for VTE prevention;
- The validation or performance benchmarking of AI tools against traditional clinical prediction scores or expert assessments;
- The practical challenges or ethical considerations of deploying AI in thrombosis care.

### 2.4. Data Organization and Synthesis

Extracted insights were organized thematically, focusing on major domains such as predictive modeling, imaging analysis, natural language processing, and wearable data integration. Rather than aggregating quantitative results for meta-analysis, we performed a qualitative synthesis to highlight key trends, methodological approaches, reported outcomes, and implementation challenges across the reviewed studies.

### 2.5. Scope and Limitations

This review aims to provide a broad, expert-level overview of the field, acknowledging that it does not capture all published studies exhaustively nor apply systematic quality scoring frameworks. The emphasis is on narrative integration, identifying both current evidence and future research directions at the intersection of artificial intelligence and VTE prevention.

## 3. Artificial Intelligence Applications in Venous Thromboembolism: Clinical Synthesis and Critical Appraisal

This section provides a structured narrative synthesis of current artificial intelligence applications in venous thromboembolism, critically examining their methodological foundations, clinical relevance, and translational readiness.

### 3.1. Predictive Modeling for VTE Risk Stratification

One of the most prominent applications of AI in VTE prevention is predictive modeling, in which ML algorithms are used to identify patients at increased thrombotic risk before the occurrence of clinical events [14]. Studies employing supervised learning approaches, including logistic regression, random forests, gradient boosting machines, and support vector machines, have demonstrated superior predictive performance compared with conventional clinical risk scores, such as the Wells and Caprini scores [15].

Key predictors incorporated into these models include patient demographics, comorbid conditions, recent surgical procedures or hospitalizations, laboratory parameters, including D-dimer levels, and medication profiles [16]. Several studies have reported AUC values exceeding 0.85, with AI-based models bring certain benefits traditional approaches particularly in complex patient populations, such as individuals with malignancy or those in the postoperative setting [17].

Notably, there is an emerging shift toward multimodal models that integrate structured EHR data with dynamic inputs, such as vital signs, indicating that real-time risk prediction may become feasible in routine clinical practice [18]. Nevertheless, many proposed models are still constrained by reliance on single-center datasets and a lack of robust external validation.

### 3.2. Advances in AI for Predicting and Preventing Venous Thromboembolism Events

Recent years have witnessed a substantial increase in research applying AI techniques to the prediction and prevention of VTE [19]. A wide range of ML models, DL architectures, and AI-based clinical decision support systems have been developed and evaluated across diverse patient populations and clinical settings [20]. These studies seek to enhance traditional risk assessment approaches by leveraging large-scale datasets, identifying complex patterns, and enabling more personalized risk prediction.

Table 1 provides a synthesized overview of key studies identified in the literature, summarizing their clinical focus, the AI methodologies employed, the study population sizes, and the principal findings related to predictive performance or clinical outcomes.

As summarized in Table 1, AI applications in VTE research encompass a broad spectrum of clinical scenarios, ranging from population-level risk prediction to specialized surgical cohorts and strategies targeting hospital-acquired VTE. Reported performance metrics, including AUC and overall predictive accuracy, consistently indicate that AI-based models have the potential to complement established clinical assessment tools. Nevertheless, despite these encouraging findings, many investigations remain limited to proof-of-concept designs or single-center analyses, showing the need for large-scale validation studies and robust real-world implementation.

### 3.3. AI in Medical Imaging for VTE Detection

In the diagnostic domain, DL, particularly convolutional neural networks, has been extensively applied to medical imaging tasks related to VTE. These applications include the interpretation of CT pulmonary angiography for pulmonary embolism and compression ultrasound imaging for deep vein thrombosis [27].

Recent studies have demonstrated that convolutional neural network–based models can achieve diagnostic sensitivities and specificities comparable to those of experienced radiologists, with reported accuracies exceeding 90% in large validation datasets [28,29,30]. Notably, several research groups have developed assistive AI tools designed to highlight suspicious regions on imaging examinations, functioning as “second readers” to support radiologists, enhance diagnostic performance, and reduce interpretative fatigue.

Despite these advances, important challenges remain, including the requirement for large, high-quality annotated imaging datasets and the risk of algorithm overfitting to specific scanner types or imaging protocols, which may limit generalizability across institutions.

### 3.4. Natural Language Processing for Risk Extraction from Unstructured Data

A comparatively underexplored yet increasingly important area involves the application of NLP to extract clinically relevant information from unstructured text sources, including physician notes, discharge summaries, and radiology reports [31]. NLP systems, particularly those based on advanced transformer architectures such as BERT, are capable of identifying risk factors—including recent immobilization, a history of malignancy, and prior thrombotic events—that are frequently absent from structured datasets [32].

Several studies have demonstrated that the integration of NLP-derived features into predictive models enhances VTE risk stratification by capturing contextual information not represented within discrete EHR fields [33,34,35,36,37]. Nonetheless, NLP applications remain largely in the developmental stage and face persistent challenges related to data privacy, heterogeneity in clinical documentation practices, and the need for language-specific model adaptation [38].

### 3.5. Integration of Wearable and Multimodal Data

Emerging research has begun to investigate the integration of wearable device–derived data, including mobility patterns, heart rate variability, and oxygen saturation, with conventional clinical information to enable dynamic assessment of VTE risk [39]. Time-series modeling approaches, such as RNNs and LSTM networks, have been proposed to analyze continuous physiological data streams and to identify early indicators of clinical deterioration or prothrombotic states [40].

Although these approaches are promising, the incorporation of wearable data into VTE risk assessment remains largely experimental, with limited real-world deployment. Major barriers include the lack of data standardization, susceptibility to signal noise, challenges related to patient adherence, and logistical difficulties associated with integrating wearable-derived metrics into existing clinical decision support systems.

### 3.6. Performance Comparison and Clinical Benchmarks

Across application domains, AI-based models generally demonstrate superior predictive performance compared with conventional clinical tools. Reported AUC values typically range from 0.80 to 0.92, with gains in sensitivity and specificity varying according to the dataset and specific clinical use case [41]. Nevertheless, many proposed models still require rigorous external validation, real-world evaluation, and direct comparison within established clinical workflows to substantiate their incremental value in routine practice [42]. Table 2 below shows an overview of the existing AI applications.

AI applications in VTE prevention encompass multiple specialized domains, each characterized by distinct technologies and varying levels of methodological maturity. In predictive modeling, ML approaches such as random forests, gradient boosting machines, and support vector machines have demonstrated substantial improvements in risk prediction, frequently achieving AUC values between 0.85 and 0.90 [47]. These models complement traditional clinical risk scores by capturing complex, nonlinear relationships within EHR data [47]. In the context of imaging analysis, DL techniques, particularly convolutional neural networks, have achieved diagnostic accuracies exceeding 90%, comparable to expert radiologists in the detection of pulmonary embolism and deep vein thrombosis using CT or ultrasound imaging [48].

Despite these advances, both predictive and imaging-based approaches face important limitations, including the need for robust external validation, susceptibility to overfitting, and the requirement for large, high-quality annotated datasets to ensure generalizability across diverse patient populations.

## 4. Translational Perspectives on Artificial Intelligence in Venous Thromboembolism Prevention

A key distinguishing feature of this review is the explicit conceptual separation of AI applications for risk prediction, diagnostic detection, and prevention strategies, recognizing that these domains entail distinct evidentiary standards, validation requirements, and barriers to clinical adoption. Furthermore, we synthesize evidence across multiple data modalities to highlight how multimodal integration may enable dynamic and personalized VTE risk assessment.

Artificial intelligence applications in venous thromboembolism should be conceptualized within three clinically distinct domains: risk prediction, diagnostic detection, and prevention-oriented clinical decision support. Although often discussed collectively, these use cases differ substantially in their objectives, evidentiary standards, and implementation challenges. Risk prediction models aim to identify patients at increased likelihood of developing VTE prior to clinical manifestation and primarily support stratification and prophylaxis decisions. Diagnostic AI systems focus on the detection or confirmation of established events, typically through imaging or natural language processing, and therefore require higher accuracy thresholds and regulatory scrutiny. In contrast, prevention-oriented workflows integrate predictive outputs into electronic health record–based decision support systems, where effectiveness depends not only on model performance but also on workflow integration, clinician engagement, and demonstrable impact on patient outcomes.

VTE remains a leading cause of preventable morbidity and mortality, despite the availability of established clinical prediction tools, biomarkers, and imaging modalities. The integration of AI into diagnostic and preventive workflows represents a paradigm shift, with the potential to improve risk stratification, enhance diagnostic accuracy, and support more personalized thromboprophylaxis strategies [49]. This review synthesizes the current landscape of AI applications in VTE prevention, emphasizing recent advances, persistent methodological challenges, and future opportunities for clinical translation [50].

### 4.1. Clinical Advantages of AI in VTE Prevention

**Figure 3:**
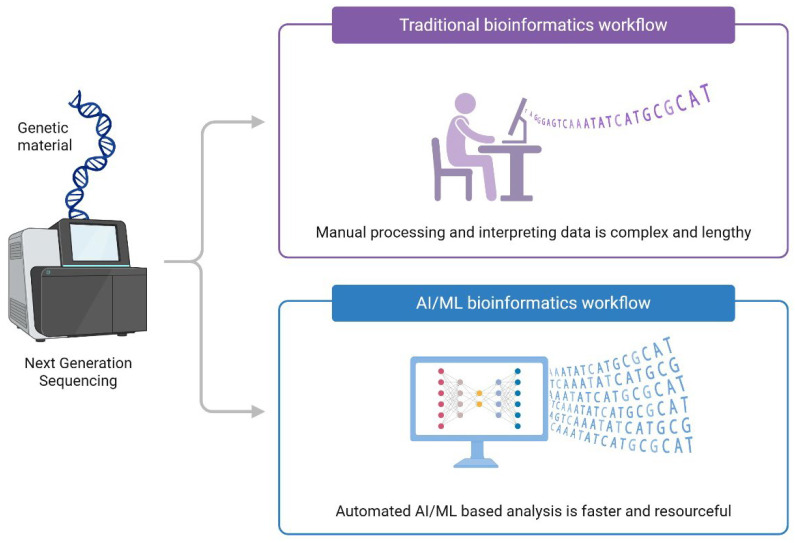
Comparative workflow: manual bioinformatics vs. AI-driven bioinformatics systems. Created in BioRender. Crisan, D. (2026) [3].

AI-based models, particularly ML and DL systems, offer several advantages over conventional clinical risk assessment tools. In contrast to traditional scores that depend on a limited number of predefined variables, such as the Wells and Caprini scores [51], AI algorithms are capable of processing large, heterogeneous, and multimodal datasets, thereby capturing complex, nonlinear interactions among multiple risk factors. Figure 3 shows a comparative workflow of the traditional and AI methods.

This capability enables more nuanced and individualized risk stratification, with the potential for earlier identification of patients at high risk who may benefit from targeted thromboprophylaxis strategies [52]. Moreover, AI-based tools can adapt and improve over time by incorporating additional data streams, including wearable device outputs, genomic information, and real-time vital sign monitoring, thereby extending their predictive capacity beyond static risk assessment scores [53]. In the diagnostic setting, DL models have demonstrated high sensitivity and specificity in the interpretation of CT pulmonary angiography and ultrasound examinations, with the potential to reduce diagnostic delays and interobserver variability.

AI has the potential to enhance VTE prevention by supporting, rather than replacing, clinical decision-making. When embedded within electronic health record–based clinical decision support systems, these approaches contribute to more timely and targeted prevention strategies; however, their clinical value ultimately depends on transparent implementation, workflow integration, and demonstrated impact on patient outcomes [54].

Beyond risk stratification, AI may contribute to more personalized thromboprophylaxis strategies by integrating comorbidities, bleeding risk profiles, and real-world outcome data into clinical decision-making. AI-based models could assist clinicians in identifying patients most likely to benefit from preventive anticoagulation while avoiding unnecessary treatment in low-risk or high-bleeding-risk individuals [55].

### 4.2. Challenges in Model Development and Validation

Despite these encouraging developments, several challenges continue to limit the widespread adoption of AI in VTE prevention. One of the principal concerns relates to data quality and representativeness, as many models are developed using single-center or retrospective datasets that may not generalize to broader patient populations because of variations in demographics, clinical practices, and data acquisition methods [52].

Overfitting remains a significant methodological issue, particularly in small or imbalanced datasets, where models may demonstrate strong internal performance yet fail to maintain accuracy during external validation. Although techniques such as cross-validation and bootstrapping can partially address this limitation, external validation using independent datasets and prospective clinical trials remains the gold standard for assessing model robustness and clinical utility [50].

Model interpretability represents another critical barrier to implementation. Many high-performing AI models, particularly deep neural networks, operate as “black boxes,” limiting clinicians’ ability to understand the rationale underlying generated predictions [56]. This lack of transparency raises concerns regarding clinician trust, ethical accountability, and regulatory approval, especially in high-stakes clinical scenarios such as anticoagulation decision-making.

### 4.3. Integration into Clinical Practice

The successful integration of AI tools into routine VTE prevention requires not only robust algorithmic performance but also carefully designed implementation strategies. Clinical decision support systems must be seamlessly embedded within EHR platforms, delivering actionable insights at the point of care while minimizing alert fatigue and avoiding disruption of established clinical workflows [57].

Equally important is clinician training and engagement to ensure that AI-generated outputs are interpreted appropriately and used to complement, rather than replace, clinical judgment. Evidence from pilot implementations and human–AI collaboration studies suggests that hybrid approaches, in which clinicians and AI systems operate in tandem, achieve superior outcomes in complex diagnostic and decision-making environments [58].

Furthermore, ethical and legal frameworks must evolve to address challenges related to algorithmic bias, data privacy, and regulatory oversight. Ensuring that AI models are transparent, explainable, and equitable across diverse patient populations is essential for safeguarding patient safety and maintaining public trust [59].

Despite increasing interest in AI applications for VTE prevention, the current evidence base remains limited in terms of external validation and prospective evaluation. Most published studies are retrospective, single-center analyses that primarily assess model performance rather than clinical effectiveness, with relatively few undergoing validation in independent populations [60].

There is a near absence of prospective, randomized, or implementation studies demonstrating improved patient outcomes with AI-guided VTE prevention strategies. Consequently, existing evidence should largely be considered proof-of-concept, highlighting technical feasibility rather than established clinical benefit [61].

A distinguishing contribution of this review is the structured separation of predictive, diagnostic, and prevention-oriented AI applications and their contextualization within real-world VTE prevention workflows, thereby shifting the focus from algorithmic performance alone to translational readiness and clinical implementation.

Despite the growing body of literature on artificial intelligence in VTE prevention, the overall level of clinical evidence remains limited. The majority of published studies are retrospective, single-center analyses primarily focused on model discrimination rather than patient-centered outcomes. External validation across diverse healthcare systems is still insufficient, and only a small number of investigations have assessed real-world implementation. Importantly, there is a near absence of large prospective, randomized, or pragmatic implementation studies demonstrating that AI-guided risk stratification or decision support systems translate into measurable reductions in VTE incidence, bleeding complications, or mortality. Consequently, much of the current evidence should be regarded as proof-of-concept, reflecting technical feasibility rather than established clinical effectiveness.

Thus, AI holds substantial potential to transform VTE prevention by enabling earlier, more accurate, and more personalized risk stratification. However, translating this promise into clinical practice requires overcoming persistent challenges related to data quality, external validation, interpretability, and workflow integration. Continued interdisciplinary collaboration among clinicians, data scientists, engineers, and policymakers will be critical to leveraging AI technologies to advance thrombosis care and improve patient outcomes on a global scale.

## 5. Conclusions

AI is rapidly transforming the landscape of VTE prevention by providing innovative tools for risk prediction, diagnostic support, and personalized thromboprophylaxis. By leveraging ML, DL, and NLP techniques, AI-based models are able to process complex, high-dimensional datasets, identify latent patterns, and generate predictive insights that extend beyond the capabilities of traditional clinical risk scores.

The existing body of evidence highlights the substantial potential of AI to improve VTE-related outcomes through earlier detection, more efficient resource utilization, and enhanced clinical decision support. High-performing models have been developed across multiple domains, including EHR data analysis, medical imaging interpretation, and the integration of wearable sensor–derived data. Nonetheless, several critical limitations remain, particularly those related to data heterogeneity, insufficient external validation, limited model interpretability, and the need for robust ethical and regulatory oversight.

For AI technologies to be successfully incorporated into routine clinical practice, rigorous prospective validation, cross-institutional collaboration, and carefully planned implementation strategies are required. In parallel, sustained efforts to promote fairness, transparency, and explainability must accompany technical innovation to preserve clinician and patient trust and to maximize clinical benefit.

Although AI applications in VTE prevention remain at an early stage of translational maturity, their capacity to reshape diagnostic paradigms and improve patient outcomes is considerable. Ongoing interdisciplinary research, stringent clinical evaluation, and responsible deployment will be essential to fully realize the potential of AI in thrombosis medicine.

## Figures and Tables

**Table 1:** Overview of recent studies applying AI models for VTE.

Study & Reference	Clinical Focus	AI Methods Applied	Population (*n*)	Key Findings
Chen et al. [21]	General population: 1-year VTE risk prediction	Machine Learning model vs. Padua clinical score	159,000	ML model AUC: 0.64–0.78; showed improved discrimination in Padua (AUC: 0.54–0.65)
Chen et al. [22]	Post-gynecological laparoscopy VTE prediction	Random Forest (RF), Artificial Neural Network (ANN), Generalized Linear Regression (GLR)	489	AUCs: RF: 0.862; ANN: 0.813; GLR: 0.709
Lin et al. [23]	VTE after hysterectomy for gynecological cancer	Decision Tree (DT), Logistic Regression (LR)	1087	DT AUC: 0.950; LR AUC: 0.722
Zhou et al. [24]	DVT prediction after gastric cancer surgery	Multivariate Logistic Regression	693	DVT prediction AUC: 0.875
Katiyar et al. [25]	VTE risk in spine surgery patients	Six ML models, including Random Forest, Simple Logistic	63	Predictive accuracy: RF: 88.89%; Simple Logistic: 84.13%
Walsh et al. [26]	Hospital-acquired VTE (HA-VTE) prevention using AI-CDSS	AI-based Clinical Decision Support System (AI-CDSS)	19,785	46% reduction in HA-VTE incidence compared to conventional prevention protocols

**Table 2:** Overview of AI applications, performance, and challenges in VTE prevention.

Domain	AI Tools Used	Reported Performance	Key Challenges
Predictive modeling [43]	Random forests, gradient boosting, SVM	AUC 0.85–0.90, improved risk prediction	External validation, dataset generalizability
Imaging analysis [44]	CNN, deep learning	Accuracy ≥ 90%, comparable to radiologists	Need for large annotated datasets, overfitting
NLP for unstructured data [45]	Transformer models, BERT-based NLP	Improved feature extraction, better context	Documentation variability, data privacy
Wearable & Multimodal integration [46]	RNN, LSTM, time-series analysis	Experimental phase, promising signal detection	Data standardization, integration into EHR

## Data Availability

No new data was created.
